# Internet-use frequency, leisure-time exercise, and offline social interaction among older adults in China: rural–non-rural differences

**DOI:** 10.3389/fpubh.2026.1978975

**Published:** 2026-10-14

**Authors:** Zhengyuan Xu, Doheung Lee, Changliang Yan

**Affiliations:** 1 Normal Branch of Yanbian University, Yanji, Jilin, China; 2 Department of Physical Education, College of Education, Seoul National University, Seoul, Republic of Korea; 3 Seoul National University (SNU) BK21 Four: Training Program for Global Leaders in Sports Science, Seoul National University, Seoul, Republic of Korea; 4 Department of Physical Education, Northeastern University, Shenyang, Liaoning, China

**Keywords:** China, internet-use frequency, leisure-time physical exercise, offline social interaction, older adults, rural–non-rural differences

## Abstract

**Introduction:**

Internet use may be associated with more active later life, but these associations may differ between rural and non-rural settings and between exercise and social interaction. We examined whether Internet-use frequency was associated with leisure-time physical exercise and offline interaction with friends and neighbors among older adults in China.

**Methods:**

This cross-sectional analysis included 2,284 adults aged 60 years or older from the in-person 2023 Chinese General Social Survey. Weighted regression models adjusted for sociodemographic and health covariates and province; standard errors were clustered by county/district code.

**Results:**

The adjusted overall associations of Internet-use frequency with exercise (B = 0.066; standardized β = 0.062) and offline social interaction (B = 0.115; standardized β = 0.094) were small. The Internet-use frequency × rural-residence interaction for exercise was negative [B = −0.170, 95% CI (−0.256, −0.084), *p* < 0.001]: the conditional association was positive among non-rural respondents (B = 0.168) and near zero among rural respondents (B = −0.002). The corresponding interaction for offline social interaction was not statistically significant (B = −0.050, p = 0.488).

**Discussion:**

Associations between Internet-use frequency and measured offline activities varied by activity and residence. Cross-sectional, frequency-based measures do not establish causal pathways or explain the contextual difference. A weighted ordinal-logit sensitivity analysis retained the negative exercise interaction, although its evidence was weaker than in the linear model.

## 1 Introduction

For older adults in China, an active life cannot be understood simply as “moving more.” Agricultural work, household labor, grandchild care, and everyday mobility may involve substantial bodily activity, particularly in rural communities, yet these activities are conceptually distinct from deliberately engaging in leisure-time physical exercise. This distinction matters because physical exercise is not merely an individual health choice. In later-life China, opportunities to exercise are embedded in family roles, intergenerational obligations, time allocation, neighborhood relationships, and locally available spaces and programs. Recent evidence from rural China illustrates this embeddedness: grandparental childcare is widespread and is associated with older adults' physical-activity participation, while intergenerational support also shapes how rural older adults allocate time across work, housekeeping, leisure, exercise, and social activities (1, 2). These patterns suggest that understanding exercise in China requires attention to the social organization of later life rather than motivation or health knowledge alone.

### 1.1 . Digital engagement and active living in later-life China

Population aging and rapid digitalization are transforming later life in China simultaneously. Internet-based communication, mobile services, digital payments, health information, short-video platforms, and online social networks have become increasingly integrated into everyday life. For older adults, digital engagement may therefore provide more than access to information: it can expand opportunities to communicate, obtain health knowledge, coordinate activities, maintain social relationships, and participate in offline life. Yet the growing presence of digital technology does not imply that older adults obtain equivalent real-world benefits from it. Digital inequality research has consequently moved beyond the first-level divide in access and the second-level divide in skills and patterns of use toward inequalities in the tangible outcomes derived from digital engagement (3–6). A systematic review links Internet use in later life with interpersonal interaction, community resources, and social inclusion (7). The potential overlap of digital and social exclusion has also been highlighted in the context of COVID-19 (8).

This outcome-oriented perspective is particularly relevant to older adults in China because digital engagement in later life is not an entirely individual process. Older adults frequently learn applications, solve device problems, and make decisions about online activities through family relationships, especially interactions with adult children and younger family members. Recent evidence based on the China Longitudinal Aging Social Survey shows that different profiles of intergenerational support are systematically associated with the frequency, continuity, and purposes of older adults' digital-media use (9). Digital engagement in later-life China can therefore be understood as socially embedded: the resources that enable and sustain digital participation often arise within intergenerational and family relationships rather than solely from individual technological competence.

General digital engagement includes access, skills, purposes, and types of online activity. The present CGSS item measures only how frequently respondents used the Internet in the previous year. We therefore use “Internet-use frequency” for the measured predictor and reserve “digital engagement” for the broader literature. Whether and how online activity might relate to offline behavior is a conceptual question; the cross-sectional survey does not observe a transition from online exposure to later behavior.

In this study, active living is an organizing concept rather than a directly measured construct. We examine two specific outcomes: frequency of leisure-time physical exercise and frequency of offline social and recreational interaction with friends and neighbors. Neither item captures total physical activity, the quality of relationships, or the full range of active living. Comparing these outcomes asks whether associations with Internet-use frequency differ across measured activities.

### 1.2 . Physical exercise as a culturally and contextually embedded health behavior

Physical exercise deserves particular attention because it is one of the most visible behavioral expressions of active aging, but its meaning in later-life China cannot be reduced to the amount of bodily movement an individual accumulates. Physical exercise and total physical activity are not interchangeable concepts. Older adults may accumulate physical activity through occupational work, transportation, household labor, farming, or caregiving without deliberately engaging in leisure-time exercise. This distinction is especially important in rural China, where productive labor and family responsibilities can remain important components of daily life after conventional retirement age. Consequently, a low frequency of leisure-time exercise should not automatically be interpreted as physical inactivity. Chinese research has long shown the importance of distinguishing occupational, household, transport, and leisure domains when interpreting activity patterns (10–12).

This distinction also changes how exercise behavior should be interpreted in the Chinese social context. Much health-behavior research appropriately emphasizes knowledge, motivation, self-efficacy, and intention, but later-life exercise is also organized around family responsibilities and the allocation of time between personal and intergenerational roles. Older adults may provide grandchild care, undertake household tasks, contribute to family production, or receive practical support from adult children. Recent evidence among rural Chinese older adults shows that grandparental childcare is widespread and is associated with participation in physical activity, illustrating that family roles can be intertwined with older adults' activity patterns rather than merely operating as background characteristics (1). Research on rural social participation likewise shows that caring, financial, and emotional exchanges between generations are associated with distinct patterns of work, housekeeping, leisure, exercise, and social connection (2). These findings support viewing later-life exercise as socially embedded rather than as an isolated individual choice.

Physical exercise is also strongly opportunity-dependent. Digital engagement may increase awareness of exercise benefits, provide instructional content, expose older adults to peer examples, or disseminate information about nearby activities. Yet these inputs can correspond to behavior only when older adults have feasible opportunities to act on them. Chinese built-environment research demonstrates that the immediate environment matters for older adults' activity. For example, a 2024 study in Nanjing linked characteristics of urban blocks—including pedestrian connectivity, land-use features, and proximity to parks and plazas—to older adults' walking activities (13). National fitness policies have sought to expand and equalize public fitness services, but equal policy commitments do not necessarily create equivalent exercise opportunities in older adults' immediate living environments (14, 15).

Importantly, rural–non-rural comparisons should not be interpreted through a simple assumption that more infrastructure necessarily means more total physical activity. Rural older adults may perform substantial bodily activity through farming, household production, transportation, domestic work, or caregiving while simultaneously participating less often in deliberate leisure exercise. The CGSS item used in this study records exercise frequency and does not measure the full spectrum of these activity domains. What may be unequally distributed across residential contexts is therefore not bodily movement *per se*, but the opportunity to organize physical exercise as a voluntary component of later-life leisure. This distinction makes the digital question more specific: Internet use can lower informational barriers, but it cannot by itself create discretionary time, accessible exercise spaces, transport, organized programs, or relief from family responsibilities.

Accordingly, the psychologically and behaviorally relevant question is not simply whether older Internet users exercise more. It is whether digital possibilities can be enacted as feasible exercise behavior within the social and material organization of everyday life. We describe physical exercise as relatively opportunity-dependent behavioral enactment. This is an interpretive distinction, not a measured mediator. Its purpose is to clarify why similar levels of digital engagement may correspond to different exercise patterns when older adults live within different family-role arrangements and local opportunity structures.

### 1.3 . Rural–non-rural contexts and domain-specific pathways to active living

Rural–non-rural residence provides an important context in which to examine this translation. Differences between rural and non-rural China extend beyond Internet penetration. Residential settings can differ in transport, population density, public space, recreational facilities, organized services, occupational routines, and the geographic distribution of social opportunities. These conditions matter because the behavioral return to digital engagement depends on whether digitally acquired information and motivation can be acted upon locally. In non-rural communities, denser service environments, public spaces, organized programs, and transportation may make it easier to act on exercise information. In rural communities, the same digital information may encounter a different combination of family responsibilities, occupational routines, travel requirements, and locally available leisure opportunities.

These contextual differences lead to a directional expectation for physical exercise. Internet use can provide exercise information, health knowledge, peer examples, and coordination in both rural and non-rural settings, but the association between digital engagement and deliberate leisure exercise should be weaker where opportunities for enactment are more constrained or differently organized. This prediction does not assume that rural older adults are less active overall. Rather, it concerns the correspondence between digital engagement and a specific form of voluntary leisure-time exercise. The distinction is particularly important because recent Chinese research continues to show that family roles and local environments are intertwined with older adults' activity patterns (1, 13).

Offline social participation offers a theoretically informative comparison because its contextual requirements may differ. The CGSS items capture participation in social and recreational activities with friends and neighbors, making this domain particularly relevant to established local relationships. Internet use can reduce communication and coordination costs, maintain contact, circulate information, and facilitate meetings. These functions can operate through existing social ties even when formal recreational infrastructure is limited. In later-life China, where family, neighborhood, friendship, and community relationships remain important organizational contexts of everyday social life, digital communication may therefore complement established offline networks. Evidence from rural China showing that intergenerational support is associated with distinct social-participation patterns further underscores the relational embeddedness of later-life participation (2). Research among rural middle-aged and older adults has also examined digital inclusion and social participation in relation to subjective well-being (16).

The contrast suggests two analytically distinct forms of offline enactment. Physical exercise may represent relatively opportunity-dependent behavioral enactment because information and motivation must be matched with time, facilities, spaces, and feasible opportunities. Social participation may represent relatively relationship-embedded relational enactment because digital communication can operate through already established interpersonal networks. These are interpretive distinctions rather than directly measured mechanisms. Their value lies in generating a domain-specific expectation: rural–non-rural inequality in the offline correlates of digital engagement should be more visible for physical exercise than for social participation.

This distinction also moves the present study beyond an increasingly crowded literature linking Internet use, exercise, social interaction, and health among Chinese older adults. A recent CGSS-based study, for example, modeled social interaction and physical exercise as sequential mediators between Internet use and health status (17). Related studies have examined social participation (18, 19) and physical exercise with self-rated health (20) as potential intermediaries between Internet use and depressive symptoms. Rather than proposing another mediation sequence, we ask a different question: why might the same degree of digital engagement correspond to different forms of active living across residential contexts? By placing physical exercise at the center of the analysis, using social participation as a relational comparison, and testing rural–non-rural heterogeneity, the study shifts attention from whether Internet use has a beneficial average association to the conditions under which digital engagement corresponds to enacted offline behavior.

Against this background, the study makes three contributions. First, it shifts attention from digital access and use to active offline enactment. Second, it treats physical exercise as the focal health behavior while comparing it with a relational domain of active living, allowing the analysis to distinguish opportunity-dependent and relationship-embedded patterns. Third, it situates these associations within later-life China, where intergenerational relationships, family responsibilities, everyday labor, familiar-tie networks, and rural–non-rural opportunity structures may shape whether digital possibilities become offline action. Because these contextual mechanisms are not directly measured in CGSS (21), they motivate the hypotheses and interpretation but are not treated as empirically established mediators.

1. **H1:** More frequent Internet use is associated with more frequent leisure-time physical exercise among older adults.
2. **H2:** More frequent Internet use is associated with more frequent offline social interaction with friends and neighbors among older adults.
3. **H3:** The association between Internet-use frequency and leisure-time physical exercise is weaker among rural than non-rural older adults.

1. **RQ1:** Does rural–non-rural residence modify the association between Internet-use frequency and offline social interaction?
2. **RQ2:** After adjustment for Internet-use frequency and covariates, are leisure-time physical exercise and offline social interaction associated with depressive affect?

Figure 1 summarizes the conceptual framework and the associations examined in this study.

**Figure 1:**
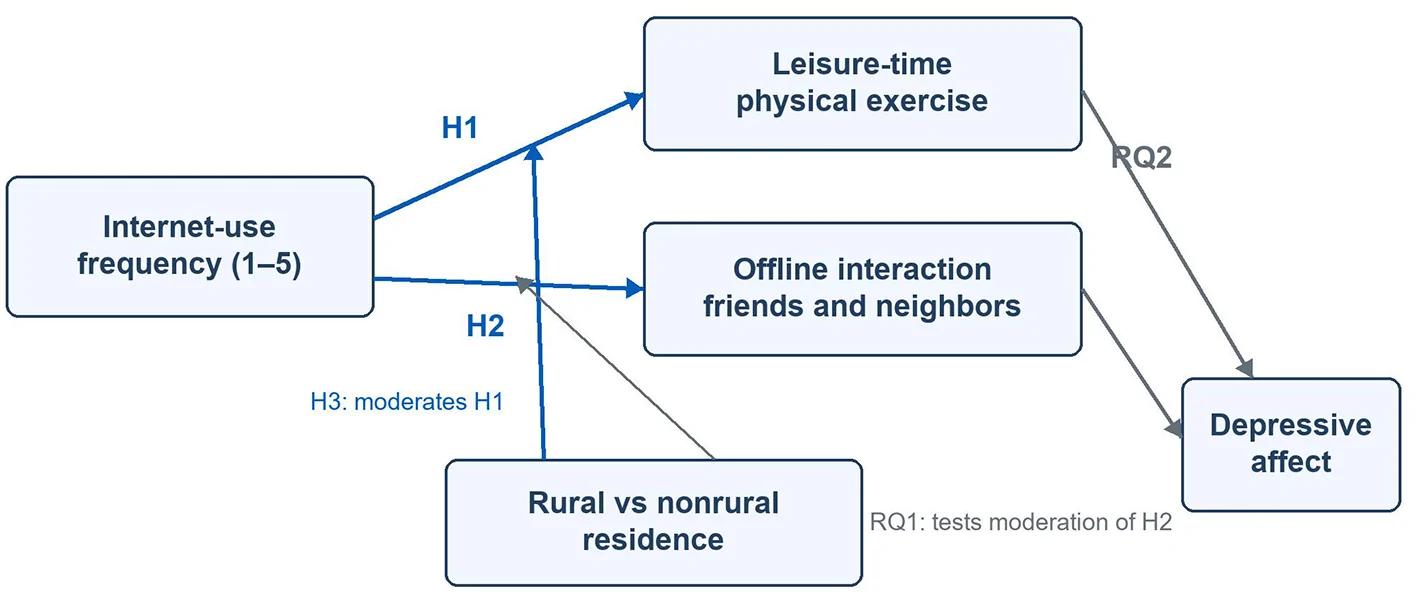
Conceptual framework and tested associations. Solid arrows denote H1 and H2; the rural-residence moderation of the Internet-use–exercise association denotes H3. The dashed moderation of the Internet-use–social-interaction association denotes exploratory RQ1. Arrows from exercise and social interaction to depressive affect denote associational RQ2 models. The diagram represents statistical associations, not temporal or causal pathways.

## 2 Materials and methods

### 2.1 Study design and data source

This cross-sectional associational study used the 2023 Chinese General Social Survey (21), a nationwide survey administered by the National Survey Research Center at Renmin University of China. The public file contains 11,326 respondents: 5,583 interviewed in person and 5,743 by telephone. The Internet-use and leisure-activity items required for this analysis, as well as the in-person weight and county/district identifier, were unavailable in the telephone component. We therefore restricted the analysis to in-person respondents and used the in-person weight (weight 1), rather than the combined-mode weight (weight 2). Weight 1 is the mode-appropriate sampling weight for the eligible interview component; its use does not by itself make the age-restricted complete-case sample nationally representative. The unit of analysis was the respondent, and age was approximated as 2023 minus birth year.

### 2.2 Participants

Among 5,583 respondents who completed the in-person survey, 2,353 had valid birth years from 1900 to 1963 and were aged 60 years or older. CGSS special codes for refusal, “do not know,” “not applicable,” or items not administered were treated as missing. Complete-case screening was then applied sequentially to the variables required for the primary models. Of the 2,353 age-eligible respondents, 2 were excluded for invalid Internet-use responses, 6 additional respondents for invalid physical-exercise responses, 5 for invalid neighbor-participation responses, 12 for invalid friend-participation responses, 10 for invalid depressive-affect responses, 13 for invalid rural–non-rural residence responses, and 20 for invalid household-economic-position responses. One additional respondent selected the residual “other” education category, which could not be placed in the pre-specified education categories and was excluded. Sex, marital status, self-rated health, province, county/district identifier, and survey weight contained no further invalid values among otherwise eligible cases. This sequential procedure excluded 69 respondents (2.93%) and yielded a final analytic sample of 2,284 older adults. Because missingness could overlap across variables, the numbers above are sequential rather than marginal counts and therefore sum exactly to the 69 excluded cases.

Sample-flow note. The complete-case exclusion affected 69 of 2,353 age-eligible in-person respondents (2.93%). Marginal counts of missing or invalid values among these 2,353 respondents were: Internet use 2, leisure-time exercise 7, either social-interaction item 19, depressive affect 11, residence 14, household economic position 22, and education 1; other model variables had no missing values. These variable-specific counts overlap and therefore must not be added to reproduce the sequential exclusions in Table 1.

**Table 1:** Sample-selection process.

Selection step	Retained *n*	Excluded at step
CGSS, 2023 (21) original file	11,326	–
Restricted to in-person interviews	5,583	5,743
Valid birth year (1900–1963), age ≥60	2,353	3,230
Valid Internet-use response	2,351	2
Valid physical-exercise response	2,345	6
Valid neighbor social-participation response	2,340	5
Valid friend social-participation response	2,328	12
Valid depressive-affect response	2,318	10
Valid rural–non-rural residence	2,305	13
Valid household economic position	2,285	20
Valid substantive education category	2,284	1

Negative special codes were treated as missing. Age was approximated as survey year minus birth year.

### 2.3 Measures

#### 2.3.1 Internet-use frequency

Internet-use frequency was measured with CGSS item a285, which asked about Internet use during the previous year through a mobile phone, computer, tablet, smart wearable device, or another device. Responses ranged from 1 (never) to 5 (very frequently). This single ordinal item is an indicator of general use frequency; it does not measure digital skills, access quality, specific online activities, duration, content, or purpose. Leisure-time Internet use (a3012) was examined as an alternative exposure in the reported sensitivity analysis. We interpret all estimates for a285 as associations with Internet-use frequency, not with multidimensional digital engagement.

#### 2.3.2 Leisure-time physical exercise

Leisure-time physical exercise was measured with item a3009. Responses from 1 (every day) to 5 (never) were reversed as 6 minus the original value, yielding a 1 (never) to 5 (daily) measure. The item records frequency only and excludes intensity, duration, energy expenditure, occupational activity, transport, farming, housework, and caregiving. It is therefore termed leisure-time physical exercise, not total physical activity.

#### 2.3.3 Offline social interaction

Offline social interaction was measured with items a31a and a31b, which asked how often respondents engaged in social and recreational activities with neighbors and with other friends. Original 1 (almost every day) to 7 (never) scores were reversed as 8 minus the original score and averaged. The resulting index ranged from 1 to 7, with higher scores indicating more frequent interaction (Cronbach's α = 0.69). It does not measure family contact, perceived social support, relationship quality, or loneliness.

#### 2.3.4 Depressive affect

Depressive affect was measured with item a17: “During the past four weeks, how frequently did you feel depressed or downhearted?” Original responses from 1 (always) to 5 (never) were reversed as 6 minus the original score. The resulting 1–5 indicator represents frequency of depressive affect. It is a single self-report item, not a clinical diagnosis or validated multi-item depression scale.

#### 2.3.5 Residence and covariates

Residence was measured with CGSS item a25a. Respondents classified as living in rural areas were coded rural (1), whereas those in urban centers, urban fringe/urban–rural transition areas, or towns were grouped as non-rural (0). The heterogeneous reference group is therefore described as nonrural rather than urban. Covariates were age, sex, education, partner status, household economic position, self-rated health, and province fixed effects. Education was entered categorically using the valid substantive CGSS education levels (a7a = 1–13); the single residual “other” response (a7a = 14) was excluded because it could not be ordered or assigned to a substantive educational category. Higher household-economic-position and self-rated-health scores indicate better conditions.

### 2.4 Statistical analysis

Categorical variables are reported as unweighted counts and weighted percentages; ordinal variables are summarized with weighted means, standard deviations, and ranges for description. Bivariate associations used unweighted Spearman correlations. The in-person sampling weight (weight 1) was divided by its analytic-sample mean. This constant rescaling preserves weighted point estimates and is used for numerical convenience; it does not restore representation lost through age restriction or complete-case selection. Weighted linear regressions estimated associations of Internet-use frequency with exercise and offline social interaction, with depressive affect examined secondarily. Treating five ordered response categories as equally spaced yields an interpretable average category-score contrast, but this is an approximation. Models adjusted for age, sex, education, partner status, household economic position, self-rated health, and province fixed effects. Province indicators account for between-province mean differences. Standard errors were clustered across 59 observed county/district codes to allow within-code correlation. The available files do not establish that these codes are the survey's original primary sampling units, and original strata were not incorporated; consequently, these cluster-robust intervals should not be presented as a fully design-based CGSS variance analysis. Internet use was mean-centered and interacted with rural residence (0 = non-rural, 1 = rural); conditional slopes used the full cluster-robust covariance matrix. Figure 2 shows average adjusted predictions and delta-method 95% confidence intervals. Coefficients, clustered standard errors, confidence intervals, and *p* values are reported. The public file identifies s43 as a county/district location code, but does not label it as the original primary sampling unit; no explicit stratum variable was identified in the available file. Thus, the analysis uses a cluster-robust approximation rather than a verified full complex-survey design (22, 23).

**Figure 2:**
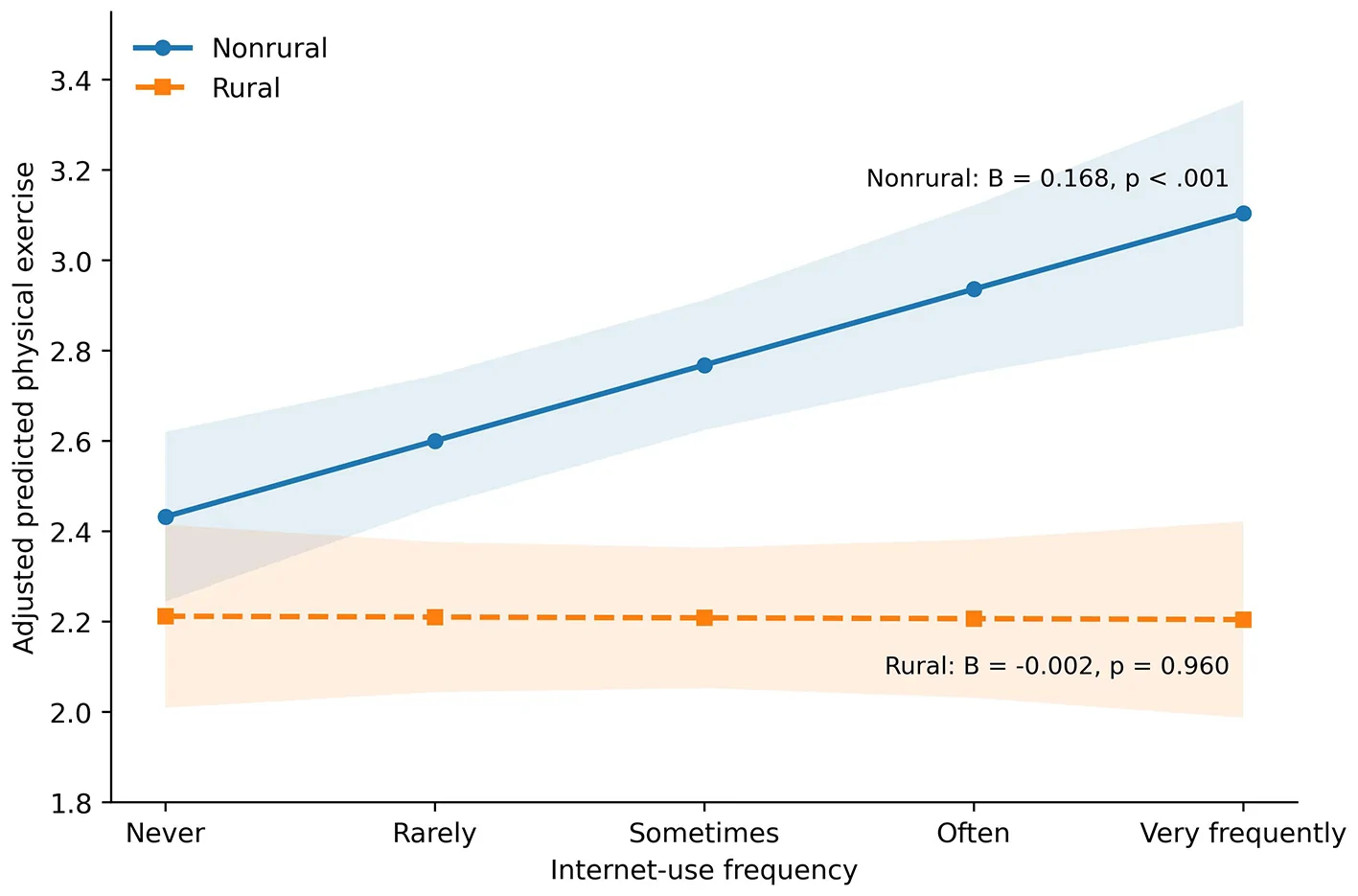
Adjusted associations between Internet-use frequency and leisure-time physical exercise by rural–non-rural residence. Lines show average model-adjusted predictions from weighted regression with province fixed effects and county/district-clustered standard errors; shaded bands show 95% confidence intervals. Non-rural slope: B = 0.168, *p* < 0.001; rural slope: B = −0.002, *p* = 0.960; Internet-use frequency × rural residence: B = −0.170, *p* < 0.001.

The exercise interaction tested the directional H3 hypothesis; the social-interaction interaction addressed exploratory RQ1. RQ2 examined adjusted associations of the two offline outcomes with depressive affect. These are associational comparisons, not tests of conversion, mediation, or temporal sequence. Cross-sectional mediation estimates need not represent longitudinal processes (24, 25). No multiplicity adjustment was applied; the exploratory and marginal findings should therefore be interpreted cautiously. To assess the equal-spacing assumption for the five-category Internet-use item, we refitted the exercise and social-interaction moderation models with category 1 (never) as reference, indicators for categories 2–5, and four category × rural-residence terms. We jointly tested the four interaction coefficients using a cluster-robust Wald χ^2^ test (4 degrees of freedom) and calculated weighted, covariate-standardized predictions for each Internet-use category in each residence group. The same complete-case sample, weight, covariates, province indicators, and county/district cluster-robust covariance estimator were retained.

Other reported sensitivity analyses assessed the Internet–depressive-affect association using leisure-time Internet use, restriction to age ≥65 years, weighted versus unweighted estimation, dichotomized Internet use, and threshold-specific logistic models for depressive-affect scores. Complete-case selection was assessed with standardized mean differences. Analyses used Python 3.12.13, pandas 3.0.1, and NumPy 2.3.5 with matrix-based weighted least-squares and cluster-robust covariance routines; the categorical-predictor Wald *p* values were obtained with SciPy. Tests were two-sided with α = 0.05. A prior cluster bootstrap was located in the analysis files but is not part of the primary-model procedure described here. For ordinal-outcome sensitivity analysis, proportional-odds logit models were fitted to the five-category exercise and depressive-affect items using the same complete cases and covariates. The in-person weight entered a weighted log-likelihood, implemented with the statsmodels 0.15.0 ordinal likelihood and SciPy 1.18.1 optimizer; county/district-clustered sandwich standard errors were used. An unweighted exercise model was also fitted. The proportional-odds assumption was not formally tested. Separately, the exploratory coefficient-product checks used 5,000 bootstrap replicates, resampling county/district codes with replacement within each province; this bootstrap was not applied to the primary exercise-interaction model.

### 2.5 Ethical considerations

This study used only de-identified CGSS (21) public-use data obtained through registration with the Chinese National Social Survey Data Archive. The authors did not recruit or contact participants, access identifiable information, or conduct additional data collection or intervention.

## 3 Results

### 3.1 Sample characteristics

The final sample comprised 2,284 adults aged 60–94 years. Weighted mean age was 69.73 years (SD = 6.71); 44.50% were women, 75.37% had a spouse or partner, and 58.41% lived in rural communities. Core-variable distributions are shown in Table 2.

**Table 2:** Sample characteristics and descriptive statistics.

Variable	*n*/weighted %	Weighted M	SD	Range
Age (years)	2,284	69.73	6.71	60–94
Women	1,205/44.50%	–	–	–
Spouse/partner	1,671/75.37%	–	–	–
Rural residence	1,145/58.41%	–	–	–
Internet-use frequency	2,284	2.71	1.61	1–5
Leisure-time physical exercise	2,284	2.40	1.71	1–5
Offline social interaction	2,284	3.86	2.01	1–7
Depressive affect	2,284	2.26	1.19	1–5
Household economic position	2,284	2.51	0.77	1–5
Self-rated health	2,284	3.00	1.16	1–5

*n* is unweighted. Percentages, means, and standard deviations use standardized weight1.

### 3.2 Bivariate associations

Table 3 presents the bivariate correlations among the core variables. Internet-use frequency correlated positively with exercise (ρ = 0.236, *p* < 0.001) and social interaction (ρ = 0.099, *p* < 0.001) and negatively with depressive affect (ρ = −0.193, *p* < 0.001). Exercise and social interaction were each negatively correlated with depressive affect.

**Table 3:** Spearman correlations among core variables.

Variable	1	2	3	4
1. Internet-use frequency	1.000			
2. Leisure-time physical exercise	0.236^***^	1.000		
3. Offline social interaction	0.099^***^	0.092^***^	1.000	
4. Depressive affect	−0.193^***^	−0.222^***^	−0.076^***^	1.000

Unweighted correlations. ^***^p < 0.001.

### 3.3 Internet-use frequency and measured offline activities

Table 4 presents the adjusted regression estimates. After adjustment, Internet-use frequency was positively associated with leisure-time physical exercise (B = 0.066, SE = 0.032, *p* = 0.036) and offline social interaction (B = 0.115, SE = 0.032, *p* < 0.001), consistent with H1 and H2. The standardized estimates were small (β = 0.062 and 0.094, respectively). Across the full four-category range of Internet-use frequency, the linear model implies a 0.264-point difference on the five-category exercise score and a 0.460-point difference on the seven-point social-interaction index, conditional on covariates; these are model-based contrasts, not expected effects of changing use. The exercise *p* value is close to 0.05 and no multiplicity adjustment was used. Internet-use frequency also had a small negative adjusted association with depressive affect (B = −0.039, *p* = 0.020). In the conditional model, neither exercise (B = −0.032, *p* = 0.074) nor social interaction (B = −0.020, *p* = 0.139) was associated with depressive affect at *p* < 0.05. RQ2 therefore yielded no clear evidence of conditional associations for the measured offline activities.

**Table 4:** Weighted regression estimates for offline resources and depressive affect.

Association	B	Clustered SE	95% CI	*p*	Std. β
Internet → exercise	0.066	0.032	[0.004, 0.128]	0.036	0.062
Internet → social interaction	0.115	0.032	[0.053, 0.177]	< 0.001	0.094
Internet → depressive affect (total)	−0.039	0.017	[−0.072, −0.006]	0.020	–
Internet → depressive affect (conditional)	−0.035	0.016	[−0.067, −0.003]	0.033	−0.047
Exercise → depressive affect	−0.032	0.018	[−0.067, 0.003]	0.074	−0.047
Social interaction → depressive affect	−0.020	0.013	[−0.046, 0.006]	0.139	−0.033

N = 2,284; 59 county/district clusters and 16 provinces. Models use standardized weight1, province fixed effects, and county/district-clustered standard errors. Outcome-model R^2^ = 0.294.

### 3.4 Rural–non-rural moderation

Table 5 presents the rural-non-rural interaction estimates and conditional slopes. The Internet-use frequency × rural-residence interaction for leisure-time exercise was negative [B = −0.170, 95% CI (−0.256, −0.084), *p* < 0.001], consistent with H3. The estimated Internet-use slope was positive in the nonrural group (B = 0.168, *p* < 0.001) and near zero in the rural group (B = −0.002, *p* = 0.960). For RQ1, the interaction for offline social interaction was not statistically significant (B = −0.050, *p* = 0.488). The depressive-affect interaction was also not significant (B = −0.001, *p* = 0.978). These results describe differences between fitted associations; they do not establish that residence changes the effects of Internet use or identify why the associations differ.

**Table 5:** Rural–non-rural moderation and simple slopes.

Outcome/term	B	Clustered SE	95% CI	*p*
Exercise: Internet slope, non-rural	0.168	0.043	[0.084, 0.252]	< 0.001
Exercise: Internet slope, rural	−0.002	0.036	[−0.073, 0.069]	0.960
Exercise: Internet × rural	−0.170	0.044	[−0.256, −0.084]	< 0.001
Social interaction: Internet slope, non-rural	0.145	0.048	[0.050, 0.239]	0.003
Social interaction: Internet × rural	−0.050	0.073	[−0.193, 0.092]	0.488
Depressive affect: Internet slope, non-rural	−0.033	0.021	[−0.074, 0.007]	0.107
Depressive affect: Internet × rural	−0.001	0.039	[−0.077, 0.075]	0.978

Internet use was mean-centered; rural = 1 and non-rural = 0. Models use standardized weight1, province fixed effects, clustered standard errors, and the same covariates as the main models. The rural exercise slope is a linear contrast based on the full cluster-robust covariance matrix.

### 3.5 Sensitivity analyses

When Internet-use frequency was entered as five categories, the joint Internet-use category × rural-residence interaction remained evident for leisure-time exercise (cluster-robust Wald χ^2^(4) = 15.53, *p* = 0.0037; *n* = 2,284; 59 county/district clusters). Weighted, covariate-standardized exercise predictions for Internet-use categories 1 through 5 were 2.36, 2.57, 2.77, 2.92, and 3.01 among non-rural respondents and 2.22, 1.93, 2.17, 2.07, and 2.25 among rural respondents. Thus, the residence difference did not depend solely on imposing a linear score on Internet use, although the category-specific patterns should not be interpreted as effects of changing use. The corresponding four-term interaction for offline social interaction was not significant (χ^2^(4) = 1.06, *p* = 0.900). Other reported sensitivity checks concerned depressive affect: estimates remained negative among adults aged ≥65 years, in unweighted models, and with leisure-time Internet use, while user/non-user and threshold-specific logistic results varied. Complete-case exclusion was 69/2,353 (2.93%); observed standardized mean differences were below |0.10|. A proportional-odds model for exercise could further assess sensitivity to treating the ordinal outcome as approximately continuous.

Ordinal-outcome models yielded a negative Internet-use frequency × rural-residence interaction for exercise under the weighted proportional-odds specification [OR = 0.880, 95% CI (0.779, 0.994), *p* = 0.039]. The corresponding unweighted model retained the negative direction but had a confidence interval spanning one [OR = 0.905, 95% CI (0.812, 1.008), *p* = 0.070]. Thus, the interaction was less precise on the ordinal scale than in the weighted linear model and its statistical significance depended on weighting. The weighted ordinal interaction for depressive affect was not significant [OR = 1.031, 95% CI (0.894, 1.187), *p* = 0.678]. The exploratory province-stratified cluster bootstrap for coefficient products gave percentile intervals of [−0.00636, −0.00004] for the pooled exercise product and [−0.00601, 0.00038] for the pooled social-interaction product; these are descriptive associations and are not evidence of causal mediation.

## 4 Discussion

### 4.1 Principal findings

The results suggest that associations between Internet-use frequency and measured offline activities differ by domain. The exercise association differed by rural–non-rural residence, whereas the social-interaction interaction was imprecise and not statistically significant. This pattern is compatible with theories of unequal opportunities to use digital information, but the data do not measure information obtained online, subsequent behavior, or local opportunities. Accordingly, we describe a domain-specific difference in cross-sectional associations rather than an observed digital-to-active-living conversion process. The weighted ordinal exercise model retained the interaction direction but produced a smaller, less precise signal; the unweighted ordinal model did not meet *p* < 0.05. This variation limits any claim of specification-independent robustness.

More frequent Internet use was associated with slightly more frequent leisure-time exercise and offline interaction with friends and neighbors in the pooled models. The estimated exercise association was positive in non-rural settings and near zero in rural settings. The interaction for offline social interaction did not provide clear evidence of a corresponding rural–non-rural difference. The two outcomes therefore should not be treated as interchangeable indicators of a single active-living process.

Depressive affect provided an important boundary condition. Internet use was weakly associated with less frequent depressive affect, but neither offline resource was conditionally associated with depressive affect at *p* < 0.05. Resource conversion therefore did not automatically imply measurable psychological benefit. This does not show that exercise or social relationships are irrelevant to mental health; rather, the specific frequency indicators and cross-sectional model did not identify them as robust correlates after mutual adjustment and covariate control. An umbrella review of exercise interventions in older adults (26) and a meta-analysis of prospective physical-activity studies in adults (27) provide broader evidence linking activity with depression outcomes.

### 4.2 From digital use to resource conversion

The findings support a distinction between digital engagement and behavioral enactment. Being online may expand access to health information, exercise demonstrations, activity announcements, communication, and coordination, but these digital inputs do not enact behavior by themselves. For physical exercise, action requires time and an actionable setting. This point is particularly important in China, where later-life exercise is embedded in family and daily-life arrangements. Recent rural evidence shows that grandparental childcare is common and related to older adults' physical-activity participation, while research on rural social participation demonstrates that intergenerational support shapes the allocation of time across family labor, housekeeping, leisure, exercise, and social activities. These studies do not explain the present interaction directly, but they provide a culturally and socially grounded reason not to treat exercise as an isolated individual choice.

The capability approach helps explain why digital engagement alone may be insufficient. A resource contributes to wellbeing only when it can be transformed into a valued functioning under relevant personal and contextual conditions (28, 29). Applied here, online information about exercise may require mobility, safe spaces, facilities, instruction, organized groups, and discretionary time. Online contact may require available partners and socially meaningful occasions. These conversion factors were not measured, so the approach serves as an interpretive aid rather than a fully tested causal model.

### 4.3 Why conversion was domain-specific

One possible explanation for the exercise interaction is variation in accessible facilities, transport, discretionary time, or family responsibilities. None of these factors was measured in the present analysis, so the interaction cannot distinguish among them. Rural respondents may also accumulate activity through farming, transport, housework, or caregiving that is not captured by the leisure-exercise item. Future studies should measure these activity domains and local conditions directly, ideally over time, before testing any contextual mechanism.

The non-significant rural interaction for offline interaction with friends and neighbors does not establish that social opportunities are equivalent across settings. The two-item index records frequency, without measuring family ties, relationship quality, support, or loneliness. Explanations involving established local networks remain hypotheses for future study.

Taken together, the two domains discourage a single hierarchy in which rural residence uniformly reduces all benefits of digital engagement. Contextual disadvantage can be specific to what an individual is trying to convert digital engagement into. Future digital inequality research should therefore model outcomes separately and measure the local opportunity structures relevant to each outcome.

### 4.4 Resource conversion and psychological benefit

The weak negative association between Internet use and depressive affect is directionally consistent with prior Chinese studies (30–33), but its magnitude was small and its significance varied when Internet use was dichotomized or the depressive-affect threshold changed. General Internet frequency aggregates potentially beneficial and harmful uses. Reverse selection is plausible: better health and mood can enable both online and offline engagement, while low mood or functional limitation can constrain them (34).

A longitudinal study of retired older adults in the United States also reported an inverse association between Internet use and depression (35). Longitudinal Chinese studies have examined social participation or group engagement in relation to depressive symptoms across retirement (36, 37). Other longitudinal analyses have linked social participation with subsequent depressive symptoms (38) and examined reciprocal associations between participation and depressive symptoms (39).

Why did offline resource participation not translate into a clear psychological correlate? First, frequency is not quality. Exercise intensity, duration, enjoyment, autonomy, and social meaning may matter more than frequency alone. Likewise, frequent contact can coexist with low support, conflict, or loneliness. A systematic review likewise emphasizes the importance of qualitative aspects of social relationships when considering late-life depression (40). Second, psychological benefits may accumulate over time and are poorly represented by a cross-sectional single-item outcome. Third, mutual adjustment may absorb shared variance between resources. Finally, a valued functioning need not produce detectable change in every psychological indicator. The finding refines the conversion lens: obtaining an offline resource is analytically distinct from realizing a downstream mental-health benefit.

### 4.5 Implications for research and practice

The findings support evaluating digital-inclusion initiatives with specific offline outcomes, including leisure-time exercise, while measuring the local conditions that may affect participation. For rural and non-rural programs alike, future intervention studies could test whether accessible exercise opportunities, transport, group activities, or family support modify outcomes. The present observational analysis cannot determine which intervention components work or whether changes in Internet use would alter exercise. Low leisure-exercise frequency should not be interpreted as low total physical activity.

Studies of social initiatives should measure relationship quality and support as well as contact frequency. Program recommendations about digital skills, alternative service channels, and mental health remain plausible considerations, but they were not evaluated by these CGSS variables and should be tested separately.

### 4.6 Strengths and limitations

Strengths include recent in-person CGSS data, explicit separation of leisure-time exercise from total physical activity, a graded Internet-use item, transparent sample selection, and reported covariate-adjusted sensitivity checks for depressive affect. Province fixed effects and county/district-clustered standard errors address specific sources of confounding and within-code dependence, subject to the survey-design limitations described above.

Several limitations qualify the findings. The cross-sectional design does not establish temporal order or causality; reverse or reciprocal associations are possible. Internet-use frequency is a single ordinal item and does not measure the broader construct of digital engagement. The categorical-predictor sensitivity analysis reduces dependence on an equal-spacing assumption for Internet use, but the primary linear model still approximates five-category exercise frequency as continuous; an ordinal-outcome analysis remains desirable. Exercise frequency does not capture duration, intensity, total volume, or work, transport, farming, domestic, and caregiving activity. Offline social interaction is limited to activities with friends and neighbors, and depressive affect is a single self-report item rather than a diagnostic measure. Local exercise facilities, transport, caregiving burden, discretionary time, and digital-use purposes were not measured, so contextual explanations remain hypotheses. The non-rural category combines several types of settlement. Weighting cannot remove selection introduced by restriction to in-person respondents aged 60 or older and complete cases, although only 2.93% of age-eligible respondents were excluded at the complete-case stage. Original survey primary sampling units and strata were not verified in the available files, limiting design-based variance claims. The proportional-odds assumption was not formally evaluated, and the weighted ordinal result was close to *p* = 0.05 while the unweighted ordinal result was not. The strength of evidence for the interaction therefore varies with the outcome model and weighting choice.

## 5 Conclusion

Among 2,284 in-person CGSS (21) respondents aged 60 years or older, Internet-use frequency was weakly associated with more frequent leisure-time exercise and offline interaction with friends and neighbors. The adjusted Internet-use–exercise association was positive among non-rural respondents and near zero among rural respondents; the social-interaction interaction was not statistically significant. The exercise interaction remained evident when Internet-use frequency was modeled categorically. Further work should test the ordinal exercise outcome and directly measure local opportunities. These cross-sectional associations do not establish that Internet use causes exercise or identify a mechanism for the rural–non-rural difference. The ordinal sensitivity analysis retained the negative interaction direction, but its precision varied by weighting; replication remains necessary.

## Data Availability

The de-identified CGSS 2023 public-use data and resident questionnaire are available through registration with the Chinese National Social Survey Data Archive, subject to the archive's terms of use: https://www.cnsda.org/index.php?r=projects/view&id=11256439.
